# A Reliable Data Transmission Model for IEEE 802.15.4e Enabled Wireless Sensor Network under WiFi Interference

**DOI:** 10.3390/s17061320

**Published:** 2017-06-07

**Authors:** Prasan Kumar Sahoo, Sudhir Ranjan Pattanaik, Shih-Lin Wu

**Affiliations:** 1 Department of Computer Science and Information Engineering, Chang Gung University, Taoyuan 33302, Taiwan; pksahoo@mail.cgu.edu.tw; 2 Department of Cardiology, Chang Gung Memorial Hospital, Linkou 33305, Taiwan; 3 Department of Electrical Engineering, Chang Gung University, Taoyuan 33302, Taiwan; d0121009@stmail.cgu.edu.tw; 4 Department of Electrical Engineering, Ming Chi University of Technology, New Taipei City 24301, Taiwan

**Keywords:** superframe, IEEE 802.15.4e, wireless sensor networks, performance analysis

## Abstract

The IEEE 802.15.4e standard proposes Medium Access Control (MAC) to support collision-free wireless channel access mechanisms for industrial, commercial and healthcare applications. However, unnecessary wastage of energy and bandwidth consumption occur due to inefficient backoff management and collisions. In this paper, a new channel access mechanism is designed for the buffer constraint sensor devices to reduce the packet drop rate, energy consumption and collisions. In order to avoid collision due to the hidden terminal problem, a new frame structure is designed for the data transmission. A new superframe structure is proposed to mitigate the problems due to WiFi and ZigBee interference. A modified superframe structure with a new retransmission opportunity for failure devices is proposed to reduce the collisions and retransmission delay with high reliability. Performance evaluation and validation of our scheme indicate that the packet drop rate, throughput, reliability, energy consumption and average delay of the nodes can be improved significantly.

## 1. Introduction

The main objective of deployment of sensors in Wireless Sensor Networks (WSNs) is to monitor the environmental conditions such as pressure, temperature, sound, etc. The physical layer and medium access control of IEEE 802.15.4-enabled [1] WSN is suitable for the low-power wireless networks. The Internet of Things (IoT), where ZigBee devices play a major role, is getting popular in smart homes and smart city applications. The existing IEEE 802.15.4 MAC scheme has been modified to meet the critical requirements of IoT applications and both the slotted and unslotted medium access control schemes for the short-range communication devices with low duty cycle have been defined in the newly-modified IEEE 802.15.4e [2] standard. The superframe structure follows the slotted MAC of the IEEE 802.15.4e and is suitable for different applications in the Internet of Things (IoT).

ZigBee technology is suitable for home and industrial automation, remote control applications and provides connectivity to different sensor devices, which can provide long battery life. On the other hand, WiFi technology gives connectivity to the IoT devices more conveniently. In different applications of IoT, such as smart home, healthcare, smart industry, smart city and smart community, both WiFi and ZigBee devices are used and operated in the 2.4-GHz ISM band, which may cause interference. The conventional MAC protocols are useless in managing the disparate transmit-power levels, asynchronous time slots and incompatible PHY layers of such heterogeneous networks and needs to be improved to cope with the existing technological demands.

The star topology is preferable for IoT applications, where the coverage vicinity is small and low latency is needed. The coordinator in the star topology controls the communication by sending packets and beacons for the synchronization among devices. All devices are allowed to communicate with the PAN coordinator only after successfully association with the coordinator. However, the communications are affected due to collisions or interference with respect to the packet arrival rate of the devices. Mostly, the network collision occurs due to simultaneous channel assessment or the hidden terminal problem. Besides, interference occurs due to the presence of both WiFi and ZigBee devices when their channels overlap. The novelty of the CSMA/CA mechanism is to avoid the collision during the ongoing transmission of another device in the network, which is not hidden. However, ZigBee’s Rx/Tx switching duration is 192 μs, and the DIFS (Distributed Inter-Frame Space) of WiFi is only 50 μs. Hence, in between ZigBee’s Rx/Tx switching duration, a WiFi transmitter can easily preempt the channel access. The coexistence of these wireless devices in a network increases the interferences, collisions, delay and energy consumptions. Moreover, ZigBee packet losses and retransmissions lead to lower reliability and faster draining of the ZigBee battery. Therefore, these issues are significant in the smart home network, where both WiFi and ZigBee devices coexist.

Sensor devices in WSNs follow the IEEE 802.15.4e MAC scheme during the Contention Access Period (CAP), performing slotted CSMA/CA to access the channel to reduce the collisions. The sensor devices have buffer constraints, and the data packet is dropped when the buffer is full. Hence, the devices having more data in the buffer should get priority to access the channel so that the packet drop rate can be minimized. However, the IEEE 802.15.4e CSMA/CA mechanism provides equal priority to all devices and does not consider the buffer limitation of the devices, which might lead to buffer overflow. The collision rate in WSNs increases with respect to the number of contending devices due to the limited backoff window size. During the Contention-Free Period (CFP), sensors transmit data directly to the coordinator. However, the transmission failures may occur due to path fading and interferences in WSNs during CFP, and sensor devices buffer the data to transmit in the next superframe. Under such a circumstance, retransmission delays and the packet drop rate increase due to the buffer constraints of the devices. Hence, it is crucial to investigate the current problems and to find solutions to reduce their impact on the overall network performance.

The main contributions of our paper are summarized as follows.

- A new frame structure is designed for data transmission, which can identify and avoid the hidden terminal problem.
- A new superframe structure is designed for the transmission failure due to interference between WiFi and ZigBee devices.
- A modified channel access mechanism is proposed to reduce the packet drop rates, energy consumptions and to enhance the throughput.
- A Markov chain model is designed to analyze the performance of our proposed MAC.
- Performance analysis of our proposed methods is made to study the reliability, latency, throughput and energy consumption.

The rest of the paper is organized as follows. The overview of IEEE 802.15.4e general superframe structure and channel access mechanism are briefly reviewed in Section 2. We review the related works in Section 3. The network model and our proposed methods are presented in Section 4. We develop the analytical models in Section 5. Performance analysis of different network parameters based on our models is studied in Section 6. Simulation results are discussed in Section 7, and concluding remarks are made in Section 8.

## 2. Overview of the IEEE 802.15.4e Protocol

The IEEE 802.15.4e standard defines the Physical layer (PHY) and MAC sublayer specifications for the devices having a low data rate, low power and short-range communication, which is quite good enough for the wireless ZigBee networks. The IEEE 802.15.4e-enabled devices operate in the license-free 868∼868.6-MHz, 902∼928-MHz and 2400∼2483.5-MHz bands with a data rate of 250 kb/s to satisfy a set of applications. Two types of devices, a Full-Function Device (FFD) and a Reduced-Function Device (RFD), are present in the IEEE 802.15.4e-based network. Both star and peer-to-peer networks are supported by the IEEE 802.15.4e standard, and both topologies work in either a beacon-enabled mode or non-beacon-enabled mode. In a star topology, communication is normally made with the PAN coordinator only, whereas any device can communicate with any other one in its communication range in the peer-to-peer network.

The IEEE 802.15.4e standard defines the slotted medium access control scheme for the short-range communication devices with low data rates. There are many MAC schemes defined by IEEE 802.15.4e, such as Time Slotted Channel Hopping (TSCH), Low Latency Deterministic Networks (LLDN), Deterministic and Synchronous Multi channel Extension (DSME), Radio Frequency Identification blink (RFID) and Asynchronous Multi-Channel Adaptation (AMCA). However, the standard specifies the optional use of the general superframe structure under beacon-enabled mode for low duty cycle devices in the network as described in the following subsection.

### 2.1. General Superframe Structure

In a beacon-enabled network, the PAN coordinator periodically broadcasts the beacon that contains the information about the superframe structure. The length of a superframe is known as Beacon Interval (BI) and is defined by the parameter Beacon Order (BO).

$$BI=a\;base\;superframe\;duration\times 2^{BO}symbols,\;0\leq BO\leq 14$$

**Figure 1:**
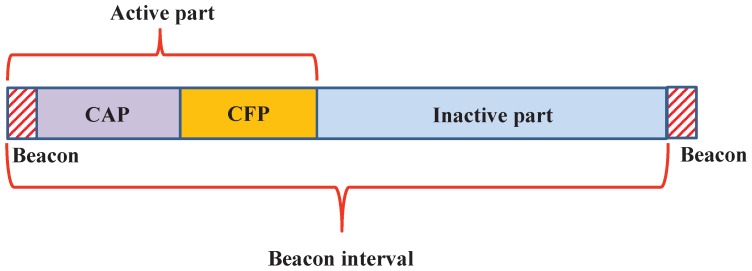
Superframe structure of IEEE 802.15.4e-enabled devices.

The BI is divided into active and inactive parts as shown in Figure 1. The active part duration is called the Superframe Duration (SD) and is defined by the parameter Superframe Order (SO).

$$\begin{array}{c} SD=a\;base\;superframe\;duration\times 2^{SO}symbols,\;0\leq SO\leq BO\leq 14 \end{array}$$

In Figure 1, the beacon duration, Contention Access Period (CAP) and CFP are present in the active part of the superframe. The superframe contains 16 superframe slots of equal duration out of which the first slot is reserved for a PAN coordinator to broadcast the beacon frame. It is used for the synchronization with the PAN coordinator and re-synchronization of the devices that go to power saving or sleep mode. In the CAP period, the devices access the channel by using the slotted CSMA/CA, whereas in CFP, the devices assess the channel by the reservation-based channel access mechanism.

### 2.2. Channel Access Mechanism

**Figure 2:**
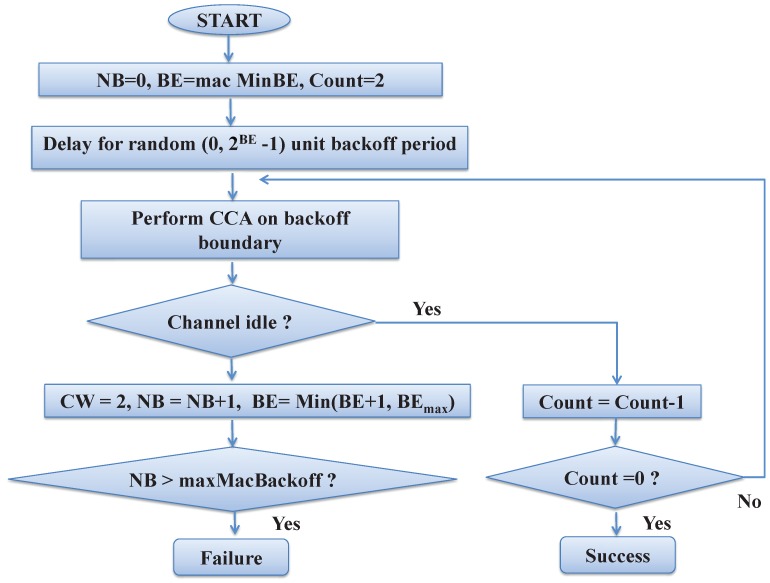
Channel access mechanism of IEEE 802.15.4e.

The devices in the network follow CSMA/CA mechanism to access the channel that is the key component in IEEE 802.15.4e MAC to communicate with the PAN coordinator. A device having data initializes the variables BE and NB, where BE is the backoff exponent and NB represents the number of times the CSMA/CA mechanism is required to be postponed before attempting the transmission. Before every transmission, the value of NB is initialized to zero. The MAC layer waits for a random backoff period in the range of $\left[0,2^{BE-1}\right]$ units as the backoff period before accessing the channel. After the backoff duration expires, the tagged device goes for two times clear channel assessment (CCA) to know the channel condition and allows transmitting data provided the channel is found idle during both CCAs. After transmitting the data, the tagged device waits for the acknowledgment, which is interpreted as the successful packet transmission. However, if the channel is found busy during any CCA, the tagged device increases the value of BE and NB by one for each channel access failure and goes for the random backoff before performing the next CCA. The maximum values of BE and NB are $BE_{max}$ and maxMacBackoff $\left(NB_{max}\right)$, respectively. The values of BE and NB are incremented for each channel access failure, and the packet is dropped by the device if the value of NB exceeds the value of $NB_{max}$. The flow chart of the IEEE 802.15.4e CSMA/CA mechanism is depicted in Figure 2.

## 3. Related Work

The IEEE 802.15.4 CSMA/CA mechanism is a deterministic approach that avoids collision by using the binary exponential backoff scheme without considering the status of the communication channel. Hence, the authors in [3] have designed one collision-aware backoff algorithm, where the nodes in the network can dynamically choose their backoff interval based on the status of the communication channel. However, the proposed algorithm cannot avoid collisions due to hidden terminal problems. To reduce the collisions, the author in [4] divide the transmission durations into a number of sub-periods based on the network environment, and one device uses only one sub-period. Though it can reduce the effects of the hidden terminal problems, it cannot provide priority to the devices with higher buffer status to access the channel first. A Time-Division Multiple Access (TDMA)-based scheme is designed in [5] for real-time data transmission to ensure collision-free transmission. The proposed time-division approach can work under ideal channel conditions, which is unrealistic. Considering the hidden terminal problem, the authors in [6] have proposed one hybrid model by combining CSMA/CA with TDMA. Though the hybrid model considers the hidden terminal problem, it requires global information of the network devices. The authors in [7] analyze the throughput of IEEE 802.15.4 MAC considering the presence of the hidden terminal problem in the network. To reduce the hidden terminal problem, the authors in [8] design one algorithm that identifies the hidden nodes by analyzing the received signal powers. However, these works focus on only ZigBee devices in the network without considering any external interferences.

Packet loss occurs due to interference when both WiFi and ZigBee channels overlap. The simple way to avoid the interferences among the devices in a network is to use different channels. However, the available channels are limited, for which the authors in [9] have designed a new MAC scheme with an increasing number of CCAs to alleviate the collisions in WiFi transmissions. However, the proposed mechanisms cannot avoid collisions due to the hidden terminal problem, and also, a significant amount of energy for CCAs is consumed in these methods. To detect the interference, the authors in [10] propose a new protocol that helps a device to switch adaptively into a safe channel. However, performance analysis in terms of latency and power consumption is not made in such adaptive channel switching. Under external interference, the theoretical analysis of throughput for WSNs has been studied in [11]. The authors in [12] have analyzed the interferences due to signal arrival direction and losses under the smart building scenario, though the reliability of the data communications is not considered. The authors in [13] propose one cooperative carrier signaling technique to avoid the interference between ZigBee and WiFi devices. However, the main drawback is that one dedicated ZigBee device should be present in the network to send the busy signal, which can increase the network cost. To analyze the performance of the coexistence of WiFi and ZigBee devices, the authors in [14] have designed the Markov model for normalized saturation throughput to ensure the quality of service and fairness. In the proposed method, both transmission failure devices and ready to transmit devices have equal priority to access the channel.

The battery power is a major constraint for ZigBee devices, and the major power consumption by the ZigBee device is due to data retransmission when there is a collision or channel error. Hence, the authors in [15] have designed one Additional Carrier Sensing (ACS) algorithm to reduce energy consumptions by avoiding further channel access through the information during the second CCA. However, the wastage of energy is possible when the second CCA is found busy due to data transmission. A new MAC protocol is designed in [16] to avoid the channel being busy due to acknowledgment packet transmission without any additional CCA. However, it cannot avoid collisions due to the hidden terminal problem. Since, one acknowledgment packet is about 22 symbols and one backoff slot is about 20 symbols, the authors in [17] propose one segmentized CCA to increase the possibilities of transmission by neglecting the channel busy condition for the shake of the ACK packet. However, it cannot avoid the end of the data packet instead of the ACK one. Apart from this, the authors in [18] design an analytical model to predict the energy consumption and throughput for the saturated and unsaturated traffics. However, these works have not considered the buffer status of the devices to improve the energy efficiency. The authors in [19] have introduced the Markov model to calculate the impact of energy consumption based on the probability of delivering a packet. A detailed analysis of the energy consumption of the Body Area Networks (BAN) with respect to packet inter-arrival time is discussed in [20]. However, the authors have not considered the external WiFi interferences with the ZigBee devices.

The authors in [21] have designed one generalized technique to investigate the performance for the slotted CSMA/CA scheme in the IEEE 802.15.4 standard. The majority of the present analytical models consider ideal channel conditions, though wireless channels are prone to burst errors. Hence, the authors in [22] have designed one three-dimensional Markov model to evaluate the performance of the IEEE 802.15.4 MAC scheme considering the burst channel errors. However, they have not considered the collisions due to the hidden terminal problem in the network. The authors have designed the time critical services in [23] and the priority-based adaptive MAC schemes for the WSNs in [24]. However, none of these schemes focus on how to enhance the reliability of the network. To  analyze the reliability, the authors in [25] design one analytical model to study the performance of MAC with the assumption that only ZigBee devices are present in the network. However, they have not considered the external network interferences.

In this paper, we not only propose a new superframe structure to avoid the hidden terminal problem in the network to enhance the reliability, but also design one channel access mechanism based on the current buffer condition of the device to reduce the packet drop rates. In addition, one analytical model is proposed to evaluate the performance in terms of throughput, delay, energy consumption and reliability considering the presence of both WiFi and ZigBee devices in a system.

## 4. Proposed Methods

In order to reduce the collisions and packet drop rates in a star topology-based wireless network, a new superframe structure and channel access mechanism are developed. Details of the network model and proposed schemes are discussed as follows.

### 4.1. Network Model

Consider a star topology-based WSN for different IoT applications such as smart healthcare, smart city and smart home, where all devices are connected to the network coordinator and follow the IEEE 802.15.4e superframe structure. It is assumed that the transmission range of the devices are equal to their sensing range. The CSMA/CA mechanism of IEEE 802.15.4e MAC is the key component, which allows the devices to access the channel uniformly. In our proposed model, all of the devices follow the CSMA/CA mechanism during the CAP period to transmit the data/guarrented time slot(GTS)request frames to the coordinator. Since power consumption is one of the major issues in WSNs, all devices go for the power saving mode after data transmission. A device that accesses the channel during the CAP period can defer transmission to a next superframe if the remaining slots are not enough.

### 4.2. Proposed Data Transmission Frame Structure

In this section, the structure of a new data frame is proposed, which can detect the hidden devices attached to the coordinator in a star topology. The main concept of the hidden terminal problem is that the carrier sensing range of a node is not large enough to detect the ongoing transmission. As per our assumption, the carrier sensing range of one device is equal to its transmission range, and a device cannot detect the ongoing transmissions by other devices due to the presence of obstacles. However, in a star topology, all devices are present within the communication range of the PAN coordinator. Hence, collision can occur only at the PAN coordinator for which the PAN coordinator does not transmit the acknowledgment to the devices.

**Figure 3:**
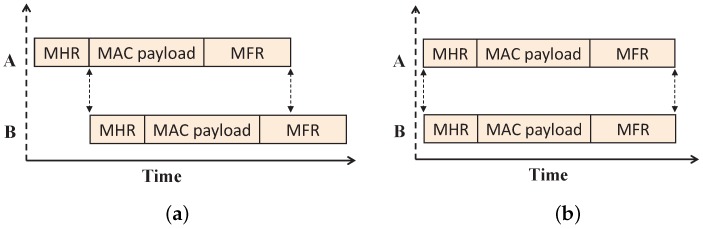
Overlapping of the data transmission frame due to the hidden terminal problem. (**a**) Partial overlapping of the data transmission frame; (**b**) full overlapping of the data transmission frame.

In WSN, when a packet is partially received, the information in the header can often be recovered and is used to identify the transmitter of that corrupted packet through the capture effects [26]. Even if the strength of two signals is the same, the radios of the sensor devices can detect and decode the undamaged signals through this capture effect. This can be only possible if the header of the colliding packet is not overlapped. Suppose two devices *A* and *B* are hidden from each other and device *B* transmits its data when the transmission of device *A* is going on. Hence, collision occurs at the PAN coordinator as their transmissions overlap fully or partially as shown in Figure 3. According to the IEEE 802.15.4e standard, the data transmission frame contains the MAC header (MHR), MAC payload and MAC footer (FHR). As shown in Figure 3a, partial overlapping occurs when device *B* transmits data after the MHR part of device *A*. The data frames overlap fully, when both devices *A* and *B* transmit simultaneously as shown in Figure 3b. The PAN coordinator can recover the sender’s address correctly from the partial frame; those are not overlapping with the subsequent frames after collision occurs due to such hidden devices.

**Figure 4:**
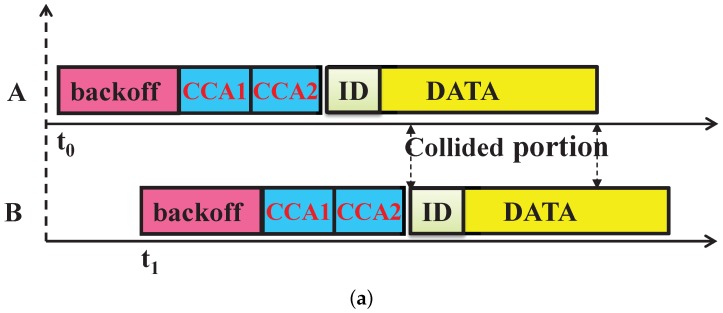

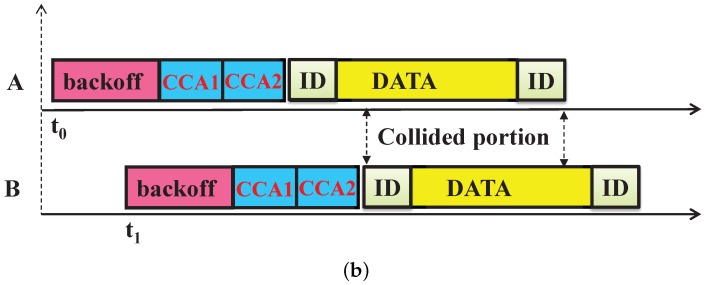
Address recovery during collisions due to hidden nodes. (**a**) Address of device Ais recovered correctly; (**b**) the address of both devices *A* and *B* is recovered correctly.

**Figure 5:**
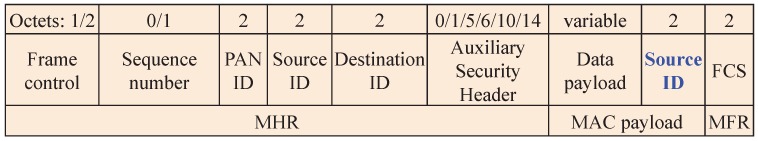
The proposed data frame structure.

For example, as shown in Figure 4a, the address of device *A* is recovered correctly [26] after the collision with hidden device *B*. Then, the PAN coordinator allocates one GTS slot to the device *A* in order to avoid subsequent hidden device collisions. However, in order to identify the other device associated with the collision (here, device *B*), we propose to add a replica of the sender device address in the rear part of the data frame as shown in Figure 5. This enables the PAN coordinator to correctly recover the address of the second device associated with the collision (here, device *B*), as shown in Figure 4b. In the sparse network, the probability that the second device has a collision with another third device is less. However, if multiple collisions occur due to hidden nodes, then the first and last device associated with the collision are correctly identified, and both devices are allotted GTS slots for data transmission. This proposed method reduces the subsequent collisions in the network by restricting the collided devices to go for further contention.

### 4.3. Proposed Superframe Structure

In the previous section, how to identify the hidden devices due to collision is discussed. It is to be noted that the hidden devices are identified only when the collided frames overlap partially and the PAN coordinator recovers the sender’s address correctly from those partially overlapped frames. However, it may happen that the collided frames overlap fully when two nodes can have the same backoff window, and therefore, the PAN coordinator cannot recover the sender address from those fully-overlapped frames. To overcome this problem due to simultaneous transmission, we propose here a newly-modified superframe structure.

**Figure 6:**
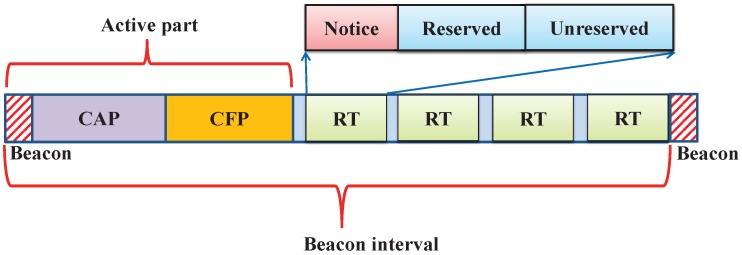
Proposed superframe structure.

As discussed in Section 2, the superframe structure contains the beacon, CAP, CFP and the inactive part. In order to reduce the delay for retransmission and to increase the reliability, we interchange the CFP and CAP parts and modify the inactive part of the superframe as shown in Figure 6. It is proposed to use this inactive part of the superframe for failure devices to improve the reliability. We partition the inactive part into a number of retransmission opportunities, and each of these retransmission intervals is again subdivided into the notice, reserved and unreserved period. Hence, our proposed superframe structure contains the beacon, CFP, CAP, notice, reserved and unreserved periods, which are defined as follows.

- Beacon period: In this period, the PAN coordinator first broadcasts the beacon in the network, and all associated devices synchronize with the PAN coordinator upon receiving the beacon successfully.
- CFP period: In this period, devices allocated with GTS slots by the PAN coordinator can transmit their data without any contention.
- CAP period: In this period, devices associated with the PAN coordinator compete with other devices, and the winner device can transmit its data. However, collisions may occur during this interval.
- Notice period: In this period, the PAN coordinator sends the acknowledgment to the devices whose data packets are successfully received. Furthermore, it informs those devices whose addresses are correctly identified during the collisions due to hidden nodes and assigns the GTS slots in the next coming reserved period.
- Reserved period: During this period, the coordinator provides a retransmission opportunity to the GTS failure devices. Besides, the devices those are correctly identified during the CAP due to the hidden terminal problem are assigned new GTS slots to transmit their data without contention.
- Unreserved period: In this period, the contended devices who have not received the acknowledgment or have not been identified by the PAN coordinator can compete among themselves to transmit their data.

**Figure 7:**
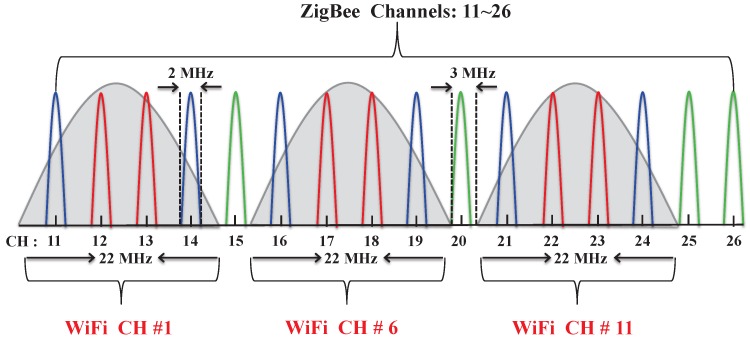
Channel overlapping between WiFi and ZigBee devices.

It is to be noted that both ZigBee and WiFi devices operate in the 2.4-GHz ISM band. The ZigBee devices use narrow band channels as compared to the WiFi’s broadband spread spectrum channels. There are 3 WiFi and 16 ZigBee channels each of 22 MHz and 2 MHz, respectively. The guard band between any two ZigBee channel is 3 MHz. Hence, as shown in Figure 7, each WiFi device’s channel overlaps with four consecutive ZigBee channels. The devices operating in those overlapping channels experience interference from the WiFi devices.

The Signal-to-Noise-plus-Interference Ratio (SNIR) is a quantity used to give theoretical upper bounds on channel capacity in wireless communication. The SINR can be determined as in [27]:

(1) $$\begin{array}{c} SINR=10\;log_{10}\left(\frac{P_{r}}{P_{n}+P_{i}}\right) \end{array}$$

where $P_{r}$, $P_{n}$, $P_{i}$ are the actual power received from the desired signal, noise power and the interferer power. The level of interferences depends on the interferer’s power, which is directly proportional to the distance from the interferer devices. Hence, the close proximity of ZigBee and WiFi devices in the overlapping channels from each other increases the interference levels. In order to avoid the interference, we suggest if channel $CH_{i}$ (for example, ZigBee channel Number 11) is used during the CAP period, then the channel $CH_{i+4}$ (i.e., ZigBee Channel 15) should be used during the retransmission period. The key features of our proposed superframe structure are that the reserved and unreserved periods are used only for the transmission failure devices to access the channel. Hence, the probability of collision and interference due to WiFi is reduced. The network coordinator is aware of the collision or interference during the CAP period. Hence, it remains active during the retransmission period. Otherwise, it goes for power saving mode. Therefore, the use of our proposed superframe structure reduces the energy consumption, delay and increases the reliability significantly.

### 4.4. New Channel Access Mechanism

Devices in WSNs follow the IEEE 802.15.4e standard channel access mechanism. In the existing CSMA/CA, all devices have equal probabilities for accessing the channel irrespective of their buffer status. However, it is more appropriate to allow the devices with higher buffer status to access the channel first so that the packet drop rate of the devices can be reduced. Besides, the CSMA/CA mechanism allows a limited contention window for which the probability of choosing the same number of random backoff periods is high. Hence, to solve this contention problem and to give priority to the devices having higher buffer status to access the channel, a new channel access mechanism is proposed. The buffer status of the devices is used to update the contention window. Initially, all devices have the same lower bound of the contention $CW_{min}=0$ window based on the IEEE 802.15.4e standard. However, nodes can have different upper bounds based on their buffer status as calculated in the following equation.

(2) $$\begin{array}{c} CW_{i}=CW_{MAX}*\frac{B_{i,Max}-B_{i,now}}{B_{i,Max}} \end{array}$$

where $CW_{i}$, $B_{i,Max}$, $B_{i,now}$ and $CW_{MAX}$ are the contention window, maximum buffer size, current buffer size of *i*-th device and the upper bound of the contention window as in the IEEE 802.15.4e standard, respectively. The IEEE 802.15.4 CSMA/CA mechanism proposes two times CCA to avoid the collision due to acknowledgment. However, in the modified superframe structure as discussed in Section 4.3, a separate time interval named the notice period is used for the purpose of acknowledgment. Hence, in our proposed channel access mechanism, we use only one CCA, which helps to decrease the energy consumption of the devices in the network. As given in Algorithm 1, let the *i*-th device attempt to transmit data during the CAP of the superframe. Accordingly, it initializes the variables NB, $CW_{i}$ and $R_{trns}$. NB is the number of times the CSMA/CA mechanism requires the delay before attempting the current transmission, which is initialized to “0” in each new transmission. $CW_{i}$ is the contention window length of the *i*-th device. $R_{trns}$ is the number of retransmission attempts. The MAC layer waits for a backoff period, i.e., a random backoff period $\left(R_{bck}\right)=random\left(0,CW_{i}\right)$ units. After the backoff period is over, a device performs its CCA provided the remaining time $\left(R_{time}\right)$ in the allotted period is sufficient to complete the transmission. Let the duration required to perform the CCA and data transmission be $T_{cca}$ and $T_{pkt}$, respectively.

Two cases may arise during the CCA, which could be either channel idle or busy. The tagged device starts transmission if the channel is found idle. Otherwise, it goes for the $random(0,T_{pkt})$ sleep period before the next attempt. The value of NB is incremented for each channel access failure up to maxMacBackoff $\left(NB_{max}\right)$. The packet is discarded, if the value of NB crosses the value $NB_{max}$ considering the channel access failure. Successful channel access does not guarantee the transmission success. The transmission failure may occur due to collisions or interference from the WiFi channels. Upon successfully receiving the data packets or decoding the addresses of the collided devices, the PAN coordinator sends the information during the notice period. It is to be noted that the upper bound of retransmission attempts due to collision or channel error is MaxFrameRetries $\left(MAX_{R_{trns}}\right)$. Each transmission failure increases the value of $\left(R_{trns}\right)$ by one. The packet is also discarded when the value of $R_{trns}$ exceeds $\left(MAX_{R_{trns}}\right)$. Otherwise, the retransmission attempts by the collided devices follow the same channel access mechanism as discussed earlier until they attain the maximum number of channel access failures or retransmission failures.
**Algorithm 1** The modified CSMA/CA mechanism
**Require:** $NB=0,R_{trns}=0,T_{cca}=8\;symbols$, packet duration $T_{pkt}$;**Ensure:** Transmission success/failure.1:Listen to the channel;2:**if** (Beacon not received) **then**3:    Wait for beacon;4:**else**5:    $CW_{i}=CW_{MAX}*\frac{B_{i,Max}-B_{i,now}}{B_{i,Max}}$;6:    $R_{bck}$ = $random(0,\;CW_{i})$;7:    **if** $\left(R_{time}\leq R_{bck}+T_{cca}+T_{pkt}\right)$ **then**8:        NB=NB+1;9:        **if** $\left(NB>NB_{max}\right)$ **then**10:           Stop due to channel access failure;11:        **else**12:           go to step 1;13:        **end if**14:    **else**15:        Access channel after $R_{bck}$ duration;16:        **if** (channel busy) **then**17:           Switch to power saving mode for $random(0,T_{pkt})$ duration;18:           NB=NB+1;19:           **if** $\left(NB>NB_{max}\right)$ **then**20:               Stop due to channel access failure;21:           **else**22:               go to step 5;23:           **end if**24:        **else**25:           Start transmission and wait for ACK;26:           **if** (ACK is not received) **then**27:               $R_{trns}=R_{trns}+1$;28:               **if** $R_{trns}\leq MAX_{R_{trns}}$ **then**29:                   Wait for next retransmission period and go to step 5;30:               **else**31:                   Stop due to transmission failure;32:               **end if**33:           **end if**34:        **end if**35:    **end if**36:**end if**

Our proposed channel access mechanism uses only one CCA and gives priority to the devices based on buffer status for which energy consumption and packet drop rate decreases significantly in the network. Again, only the collided devices can contend during the subsequent retransmission periods in different channels, which reduces the contention collision and interferences for which the reliability is increased significantly.

## 5. Analytical Models for the Proposed MAC

**Figure 8:**
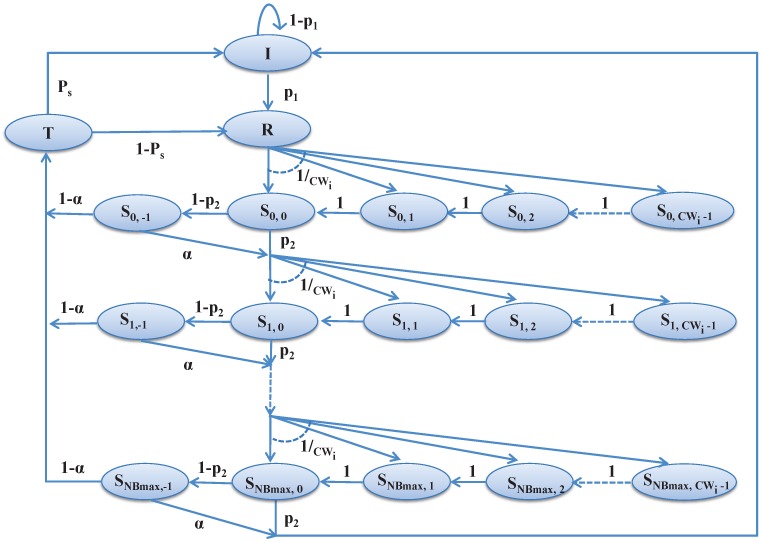
Markov chain model for the proposed channel access mechanism.

In this section, we design the Markov chain model as shown in Figure 8 to analyze the channel access scheme during the contention period for the associated devices in the network. Based on the notations given in Table 1, the analytical model is designed taking the unsaturated traffic condition.

Let *N* be the number of devices associated with a PAN coordinator in a smart home for data communications. Let s(t) and c(t) represent the backoff stage for *NB* and backoff counter for *CW* at time *t*, respectively for the stochastic processes as shown in Figure 8. Let,

$$S_{j,k}=\lim_{t\to \infty }P\left\{s\left(t\right)=j,c\left(t\right)=k\right\}$$

where *k* is the index of the backoff counter and $k\in \{-1,\ldots ,CW_{i}-1\}$, while $CW_{i}$ is the contention window for *i*-th device, *j* is the index of backoff stage and $j\in \{0,\ldots ,NB_{max}\}$, while $NB_{max}$ is the maximum number of allowable backoff stages. The state $S_{j,-1}$ represents the start of the channel access at the *j*-th backoff stage, where $j\in \{0,\ldots ,NB_{max}\}$. In our model, the states *I*, *R*, *T* stand for the idle state, ready state and transmit state, respectively. Let, $p_{1}$ be the probability that the device has a packet to transmit. After generating the packet, a device goes to the ready state *R*, chooses a random backoff and goes to the state $S_{0,k}$, where $k\in \{0,1,\ldots ,CW_{i}-1\}$. The state $S_{j,k}$ represents the backoff state.

Let α be the channel busy probability during the CCA and $p_{2}$ be the transmission deferred probability due to the current remaining time in the CAP period being insufficient to carry out the data transmission. Whenever a device finds the channel idle during CCA, it enters into the transmission state and tries to transmit packets. Let $P_{s}$ be the probability of successful packet transmission.

We can derive the transition probabilities as follows based on the proposed Markov chain model as given in Figure 8. A device after having a packet to transmit goes to the ready state, and the corresponding transition probability from the initial state to the ready state is:

(3) $$\begin{array}{c} P\left\{R|I\right\}=p_{1}. \end{array}$$

The device initially goes for the random backoff before accessing the channel and moves from the ready state to the first time backoff state $S_{0,k}$. The corresponding transition probability is deduced as follows.

(4) $$\begin{array}{c} P\left\{S_{0,k}|R\right\}=\frac{1}{CW_{i}}\;\;for\;0\leq k\leq CW_{i}-1. \end{array}$$

After reaching a backoff state, the backoff counter is decremented with probability one. The corresponding transition probability is deduced as:

(5) $$\begin{array}{c} P\left\{S_{j,k}|S_{j,k+1}\right\}=1\;\;for\;0\leq j\leq NB_{max}\;and\;0\leq k\leq CW_{i}-1. \end{array}$$

The tagged device reaches the channel sensing state provided the current remaining time in the CAP period is sufficient to carry out the data transmission. The corresponding transition probability can be derived as follows.

(6) $$\begin{array}{c} P\left\{S_{j,-1}|S_{j,0}\right\}=1-p_{2}\;\;for\;0\leq j\leq NB_{max}. \end{array}$$

However, the tagged device will go for the random backoff again, if the current remaining time in the CAP period is insufficient to carry out the data transmission. The corresponding transition probability can be derived as follows.

(7) $$\begin{array}{c} P\left\{S_{j+1,k}|S_{j,0}\right\}=\frac{p_{2}}{CW_{i}}\;\;for\;0\leq j\leq NB_{max}\;and\;0\leq k\leq CW_{i}-1. \end{array}$$

The tagged device starts data transmission after the CCA is successful. The transition probability for this case can be derived as follows.

(8) $$\begin{array}{c} P\left\{T|S_{j,-1}\right\}=1-\alpha \;\;for\;0\leq j\leq NB_{max}. \end{array}$$

However, the tagged device will go for random backoff again, if the current CCA is failed. The corresponding transition probability can be derived as follows.

(9) $$\begin{array}{c} P\left\{S_{j+1,k}|S_{j,-1}\right\}=\frac{\alpha }{CW_{i}}\;\;for\;0\leq j\leq NB_{max}\;and\;0\leq k\leq CW_{i}-1. \end{array}$$

Once the device reaches the maximum backoff attempts for transmission, the device returns back to the idle state no matter whether the transmission attempt is successful or failed. Hence, the corresponding transition probability can be deduced as follows.

(10) $$\begin{array}{c} P\left\{I|S_{NB_{max},0}\right\}=\left(p_{2}+\left(1-p_{2}\right)\alpha \right)+\left(1-p_{2}\right)\left(1-\alpha \right)P_{s}. \end{array}$$

Considering these transition probabilities, the probability of a device at state $S_{j,k}$ can be derived as follows, where $S_{j,k}$ is the stationary distribution of the Markov chain.

After the channel access failure in the current backoff state, the tagged device enters into the next backoff state, and the probability for the corresponding state is:

(11) $$\begin{array}{c} S_{j,k}=\frac{CW_{i}-k}{CW_{i}}\left[S_{j-1,0}\left\{p_{2}+\left(1-p_{2}\right)\alpha \right)\right\}];\;\;\;for\;1\leq j\leq NB_{max}\;and\;0\leq k\leq CW_{i}-1. \end{array}$$

Once the device finds the channel idle during the CCA without attaining the retry limits, it will enter into the initial backoff state, and the corresponding state probability can be deduced as:

(12) $$\begin{array}{c} S_{0,k}=\frac{CW_{i}-k}{CW_{i}}[\left\{\sum_{j=0}^{NB_{max}}S_{j,0}\right\}\left(1-p_{2}\right)\left(1-\alpha \right)\left\{\left(1-P_{S}\right)+P_{S}p_{1}\right\}\\ +S_{NB_{max},0}\left(p_{2}+\left(1-p_{2}\right)\alpha p_{1}\right];\;\;0\leq k\leq CW_{i}-1. \end{array}$$

Normally, a device defers to data transmission if the remaining time in the contention period is insufficient to complete the transmission, and we can derive the corresponding state probability as follows.

(13) $$\begin{array}{cc} S_{j,-1} & =\left(1-p_{2}\right)S_{j,0};\;\;\;for\;0\leq j\leq NB_{max}. \end{array}$$

The tagged device starts transmission after the channel access is successful during the *j*-th backoff state. Hence, the probability for the corresponding state can be deduced as follows.

(14) $$\begin{array}{cc} T & =\left(1-\alpha \right)S_{j,-1};\;\;\;for\;0\leq j\leq NB_{max}. \end{array}$$

A device enters into the idle state after completion of the data transmission or has no new packet to transmit, and the corresponding state probability is deduced as:

(15) $$\begin{array}{cc} I & =\frac{1}{p_{1}}\left(P_{s}T+\left(p_{2}+\left(1-p_{2}\right)\left(1-\alpha \right)\right)S_{NB_{max},0}\right). \end{array}$$

A device enters into the ready state whenever it has a new packet or needs to retransmit the failed packet. Hence, the corresponding state probability can be derived as follows.

(16) $$\begin{array}{cc} R & =\left(1-P_{s}\right)T+p_{1}I. \end{array}$$

The sum of the probabilities of all states should be equal to one based on the rule of the probability for which we can get the following equation.

(17) $$\begin{array}{cc} I+R+\sum_{j=0}^{NB_{max}}\sum_{i=0}^{CW_{i}-1}S_{j,i}+\sum_{j=1}^{NB_{max}}S_{j,-1}+T & =1 \end{array}$$

We can deduce the probabilities of all states by solving these equations. The probability for a device that attempts the CCA for the first time can be calculated as follows.

(18) $$\begin{array}{c} \phi =\sum_{j=0}^{NB_{max}}S_{j,0}\left(1-p_{2}\right). \end{array}$$

## 6. Performance Analysis

The bit error rate probability $P_{b}\left(\zeta \right)$ for IEEE 802.15.4 can be calculated [1] as given in the following equation.

(19) Pb(ζ)=815116∑k=216(−1)k16ke20ζ(1k−1)

where ζ is the SINR. Let, $p_{1}$ be the probability for a device having a packet for the transmission and α be the probability that the channel is found busy during CCA due to data transmission by other ZigBee devices or WiFi devices available in a smart home. Let, $\lambda_{w}$ be the Poisson packet arrival rates for WiFi devices. Therefore, the channel found busy during CCA duration ($T_{cca}$) by the ZigBee device due to WiFi transmission is $1-e^{-T_{cca}\lambda_{w}}$. Similarly, a ZigBee device found busy due to transmission of any other ZigBee device in the network is $L_{D}\left(1-{\left(1-\phi \right)}^{N-1}\right)\left(1-\alpha \right)e^{-T_{cca}\lambda_{w}}$, where $L_{D}$ is the packet length and *N* is the number of contending ZigBee devices. Therefore, the probability of finding the channel busy during CCA is given as follows.

(20) $$\begin{array}{c} \alpha =1-\left(1-L_{D}\left(1-{\left(1-\phi \right)}^{N-1}\right)\left(1-\alpha \right)\right)e^{-T_{cca}\lambda_{w}} \end{array}$$

When the channel access is failed or the current remaining duration in the contention period is insufficient for the transmission, the ZigBee device decides to go for the next backoff. Hence, the probability of going for the next backoff is:

(21) $$\begin{array}{c} P_{backoff}=p_{2}+\left(1-p_{2}\right)\alpha . \end{array}$$

### 6.1. Reliability

A transmission is always successful when exactly one ZigBee device transmits without any channel error or collision. The packet error depends on the bit error rate and the packet length. Let, $L_{Data}$ be the length of the data packet in bits, *N* be the number of ZigBee devices and $T_{pkt}$ be the ZigBee packet duration. A transmission will be successful if the ZigBee device receives the acknowledgment packet corresponding to its transmitted packet. The coordinator sends the acknowledgment packet, if exactly one ZigBee device transmits without any channel error and no WiFi device starts transmission during the ongoing ZigBee transmission. Hence, the successful transmission probability for a ZigBee device is:

(22) $$\begin{array}{cc} P_{s} & =\frac{N\phi {\left(1-\phi \right)}^{N-1}}{\left(1-{\left(1-\phi \right)}^{N}\right)}{\left(1-P_{b}\left(\zeta \right)\right)}^{L_{Data}}e^{-T_{pkt}\lambda_{w}}. \end{array}$$

The packet is dropped mainly due to retry limits or channel access failure. The channel access failure occurs when the tagged ZigBee device does not find any channel ideal during maxMacBackoff number of backoffs. Considering the Markov chain model presented in Figure 8, the probability of channel access failure can be derived as follows.

(23) $$\begin{array}{c} P_{cf}=\left\{p_{2}+\left(1-p_{2}\right)\alpha \right\}b_{m,0}. \end{array}$$

In our proposed modified superframe, separate durations are allotted for collided devices, i.e., only failure devices contend during those periods. Let $P_{s_{j}}$ be the probability of successful transmission by a ZigBee device in the *j*-th allocated retransmission duration of the proposed modified superframe. If the expected number of ZigBee devices that contend in the *j*-th retransmission period is $N_{j}$, then:

(24) $$\begin{array}{cc} P_{s_{j}} & =\frac{N_{j}\phi {\left(1-\phi \right)}^{N_{j}-1}}{\left(1-{\left(1-\phi \right)}^{N_{j}}\right)}{\left(1-P_{b}\left(\zeta \right)\right)}^{L_{Data}}e^{-T_{pkt}\lambda_{w}}. \end{array}$$

Let us assume that maxFrameRetries=r. Hence, the probability of transmission failure during all *r* attempts is:

(25) $$\begin{array}{c} P_{tf}=\prod_{j=1}^{r}\left(1-P_{s_{j}}\right) \end{array}$$

It is assumed that the generated data packets are first buffered by each device and then are transmitted to the coordinator. However, the ZigBee device has buffer constraints. Let the data arrival rate of the ZigBee devices be $\lambda_{z}$, the service rate be $\mu_{z}$ and the buffer maximum be *l* number of packets. Hence, the device can calculate its utilization rate, which is $\rho_{z}=\frac{\lambda_{z}}{\mu_{z}}$. Let $P_{bf}$ be the probability that the buffer of the ZigBee device is full, i.e., there are *l* number of packets in the buffer of the ZigBee device. Hence, the newly-generated packet will be discarded due to full buffer being:

(26) $$\begin{array}{c} P_{bf}=\frac{\rho_{z}^{l}}{\sum_{i=0}^{l}\rho_{z}^{i}} \end{array}$$

Furthermore, the reliability can be deduced as follows.

(27) $$\begin{array}{c} P_{R}=\left(1-P_{cf}\right)\left(1-P_{tf}\right)\left(1-P_{bf}\right) \end{array}$$

### 6.2. Throughput

Let *S* be the normalized throughput of a ZigBee device. The ratio of average time occupied by the successful transmission information to the interval between two consecutive transmissions is known as normalized throughput. The channel is sensed as busy, if there is at least one ZigBee device sending packets. Let $P_{tr_{j}}$ be the probability that there is at least one transmission during the *j*-th time transmission. Again, there are $N_{j}$ ZigBee devices attached to the coordinator during *j*-th time transmission, and ϕ is the probability that a ZigBee device attempts carrier sensing for the first time for transmission. Hence,

(28) $$\begin{array}{c} P_{tr_{j}}=\left(1-{\left(1-\phi \right)}^{N_{j}}\right)\left(1-\alpha \right) \end{array}$$

Therefore, the normalized throughput of a ZigBee device during the *j*-th time transmission is:

(29) $$\begin{array}{c} S_{j}=\frac{P_{tr_{j}}P_{s_{j}}L_{D}}{\left(1-P_{tr_{j}}\right)\sigma +P_{tr_{j}}P_{s_{j}}T_{s}+P_{tr_{j}}\left(1-P_{s_{j}}\right)T_{c}} \end{array}$$

where $T_{s}$ is the average time for successful transmission and $T_{c}$ is the average time that the channel is occupied due to collision. Let $L_{D}$ be the time of the payload and σ be the duration for a unit backoff slot. Since acknowledgment is transmitted separately in the notice period in our proposed superframe, the durations of these $T_{s}$ and $T_{c}$ are equal and are calculated as:

(30) $$\begin{array}{c} T_{s}=T_{c}=T_{cca}+\sigma +T_{pkt} \end{array}$$

where $T_{cca}$, $T_{pkt}$ and σ are the time duration for performing CCA, for transmitting the data and the duration of one backoff slot, respectively. Hence, the average normalized throughput of a ZigBee device is:

(31) $$\begin{array}{c} S=\frac{\sum_{j=0}^{r}S_{j}}{r} \end{array}$$

### 6.3. Delay

The network delay is defined as the total time taken for the information to travel from the source device to the destination device. It is to be noted that in our proposed modified superframe transmission, failure devices use separate retransmission period. Let $D_{j}$ be the delay that the packet is transmitted successfully at time j+1. Then,

(32) $$\begin{array}{cc} D_{j} & =T_{s}+jT_{R}+\sum_{i=0}^{j}D_{b} \end{array}$$

where $D_{b}$ is the delay that the ZigBee device found the channel idle during maxMacBackoff number of backoffs, $T_{s}$ is the average time of successful transmission and $T_{R}$ is the allocated retransmission duration due to transmission failure. Let $B_{k}$ be the event that the channel access is successfully at the (k+1)-th time provided the channel access is failed for *k*-th times. Let *B* be the event that the channel access is successful within maxMacBackoff. Hence, the expected delay for backoff during one transmission attempt is:

(33) $$\begin{array}{cc} D_{b} & =\sum_{k=0}^{m}k\frac{P_{backoff}^{k}\left(1-P_{backoff}\right)}{1-P_{backoff}^{m+1}}\left(\frac{CW_{i}-1}{2}\right) \end{array}$$

A device has to wait until the next superframe starts, if the packet is generated outside of the contention period. The packet arrival of ZigBee devices is assumed to follow the Poisson distribution with average packet arrival rate $\lambda_{z}$. Then, the waiting time is:

(34) $$\begin{array}{cc} W_{time} & =\int_{0}^{BI}\left(BI-x\right)\lambda_{z}e^{-\lambda_{z}x}dx. \end{array}$$

Propagation delay $P_{delay}$ is the amount of time it takes for one bit to travel through a one-hop destination. Let $E_{j}$ be the event that the packet is transmitted successfully at time j+1 provided the packet has been failed for *j* times. Let *E* be the event that the packet is transmitted successfully within maxFrameRetries. Considering the maxFrameRetries = *r*, the conditional probability can be derived as follows.

(35) $$\begin{array}{cc} Pr(E_{j}/E) & =\frac{P_{s_{j+1}}\prod_{k=1}^{j}\left(1-P_{s_{k}}\right)}{1-\prod_{k=1}^{r}\left(1-P_{s_{k}}\right)}. \end{array}$$

Hence, the delay can be derived as follows.

(36) $$\begin{array}{cc} D & =\sum_{j=0}^{r}Pr\left(E_{j}/E\right)D_{j}+W_{time}+P_{delay}. \end{array}$$

### 6.4. Energy

In this section, we analyze the energy consumption of the ZigBee devices in smart home applications. Here, the energy consumption is defined as the average energy consumption by a ZigBee device to successfully transmit one slot amount of data. Let us consider $P_{rx}$ and $P_{tx}$ to be the energy consumption to receive and to transmit a packet, respectively. ZigBee devices are assumed to be in the power saving state during the backoff procedure. Taking $T_{pkt}$, $T_{A}$, $T_{cca}$ as the time duration of transmitting a packet, receiving an acknowledgment and CCA duration, respectively, the average energy consumption per device can be analyzed as follows.

(37) $$\begin{array}{cc} E & =\frac{\alpha T_{cca}P_{rx}+\left(1-\alpha \right)\left\{\left(1-P_{s}\right)E_{c}+P_{s}E_{s}\right\}}{\left(1-\alpha \right)P_{s}T_{pkt}} \end{array}$$

where $E_{s}$ is the energy consumption of successful transmission and $E_{c}$ is energy consumption due to collision, which can be calculated as:

(38) $$\begin{array}{c} \left.\begin{array}{cc} E_{s} & =T_{cca}P_{rx}+T_{pkt}P_{tx}+T_{A}P_{rx}\\ E_{c} & =T_{cca}P_{rx}+T_{pkt}P_{tx} \end{array}\right\} \end{array}$$

## 7. Performance Evaluation

In this section, our proposed models are evaluated and validated by using the OMNeT++ [28] simulator. We conduct the simulation to compare the performance of our proposed scheme with IEEE 802.15.4e. For the simulation, we consider the star topology and devices are located randomly in the area of 30 × 20 m${}^{2}$ with the PAN coordinator at the center. Again, we compare our work with the IEEE 802.15.4e standard for channel access failure, throughput, reliability, average MAC delay, energy consumption and packet drop rate over the Poisson arrival rate. We validate and compare our proposed mechanisms with the IEEE 802.15.4e standard. According to the standard, simulation parameters are set as shown in Table 2. Each simulation is performed for 50 rounds, and the results are produced by taking the average of those 50 rounds.

**Figure 9:**
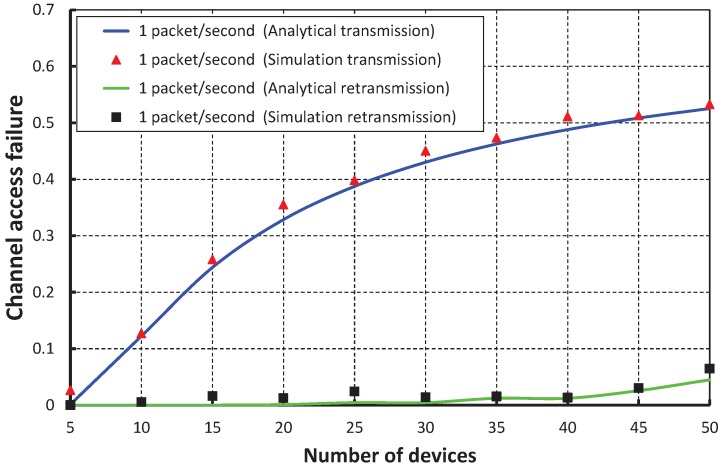
Validation of our proposed scheme for channel access failure for different numbers of devices.

**Figure 10:**
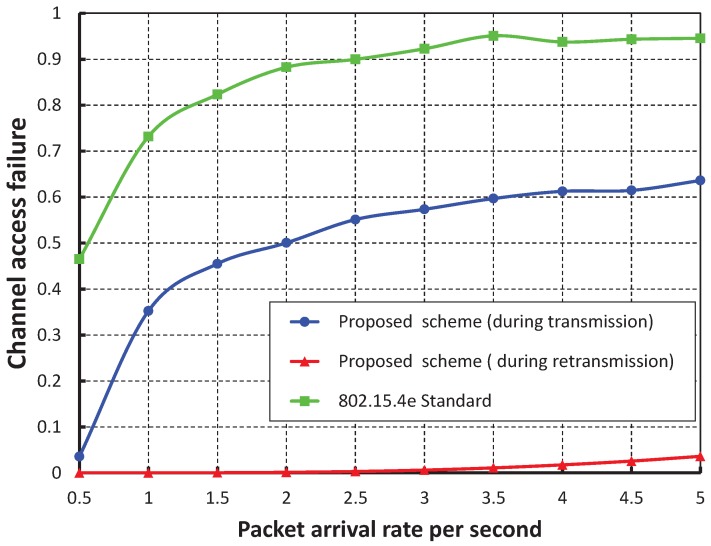
Comparison of channel access failure for different numbers of devices.

As shown in Figure 9, the number of devices are taken along the *X* axis, and the corresponding channel access failure probability is obtained as shown along the *Y* axis. It is observed that the channel access failure probability increases as the number of devices attached to a coordinator increases. However, the channel access failure following our schemes during the retransmission is negligible. In our simulation, it is considered that all devices can assess the channel during the contention access period, i.e., in the transmission period of the superframe. However, the devices that fail to assess the channel during this transmission period can access the channel during the newly-allocated retransmission period. As shown in Figure 10, when our work is compared with the IEEE 802.15.4e scheme, it is found that the channel access failure probability of our work is significantly less during both the transmission and retransmission period. Again, as shown in Figure 9, it is observed that our simulation result matches the analytical one.

**Figure 11:**
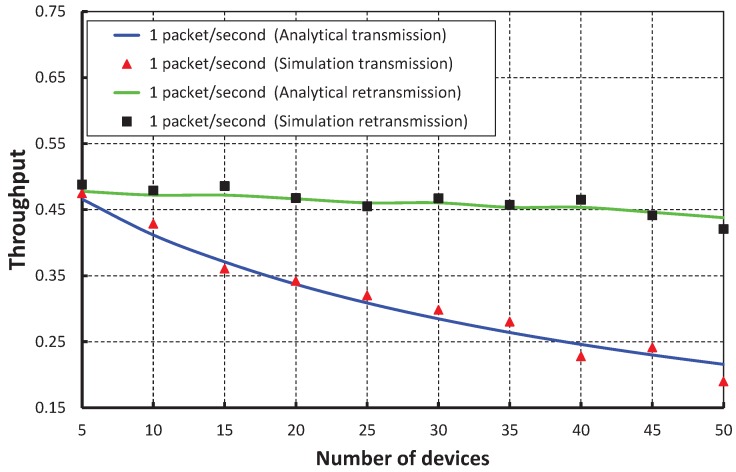
Validation of the throughput of our proposed scheme for different numbers of devices.

**Figure 12:**
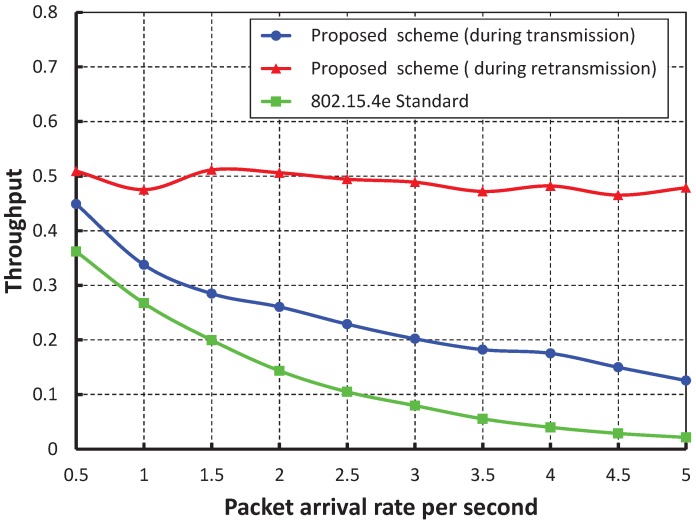
Comparison of the throughput of our proposed scheme with the IEEE 802.15.4e standard.

The throughput corresponding to different numbers of devices is depicted in Figure 11, and it is observed that the throughput decreases as the number of devices attached to a PAN coordinator increases irrespective of their packet arrival rate. When the packet arrival rate is one packet per second and five devices are attached to the coordinator, the normalized throughput is 0.46. It is observed that the throughput of our work is higher during both transmission and retransmission, which is reflected in Figure 12, when we compare our work with the IEEE 802.15.4e standard.

**Figure 13:**
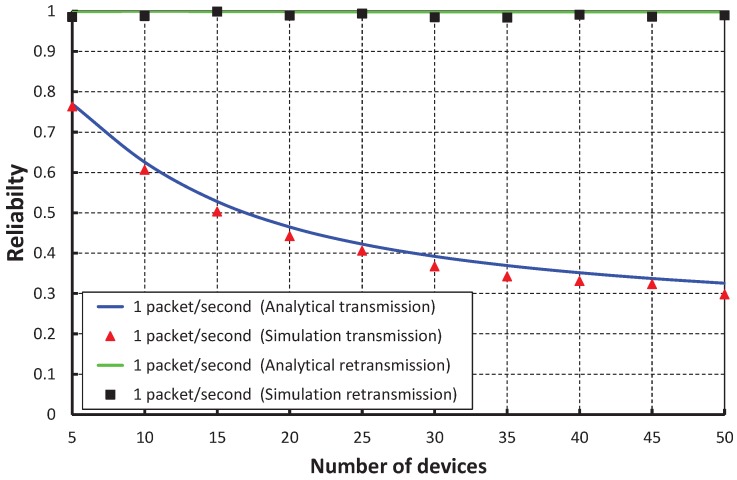
Validation of the reliability of our proposed scheme for different numbers of devices.

**Figure 14:**
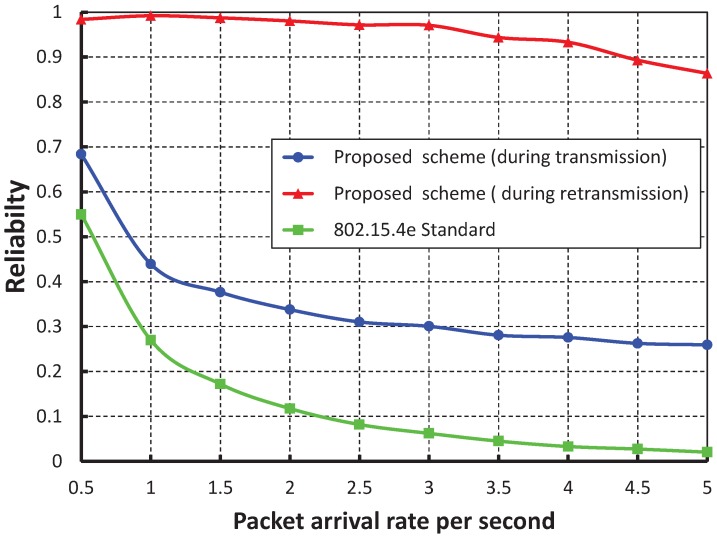
Comparison of the reliability of our proposed scheme with the IEEE 802.15.4e standard.

Figure 13 compares the reliability of data transmission with respect to different numbers of devices. When we consider the packet arrival rate as one packet per second, it is observed that the reliability decreases, along with the number of devices attached to a PAN coordinator increases. From Figure 14, it is observed that the reliability of our work is higher than the IEEE 802.15.4e scheme, and the simulation results of our scheme match with the analytical one.

**Figure 15:**
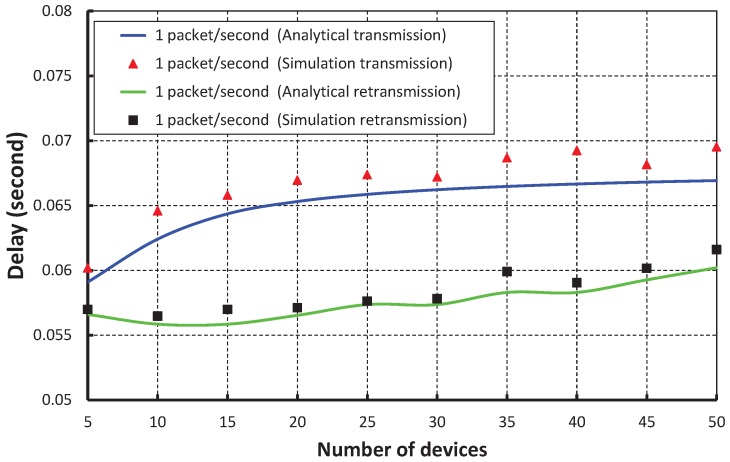
Validation of delay of our proposed scheme for different numbers of devices.

**Figure 16:**
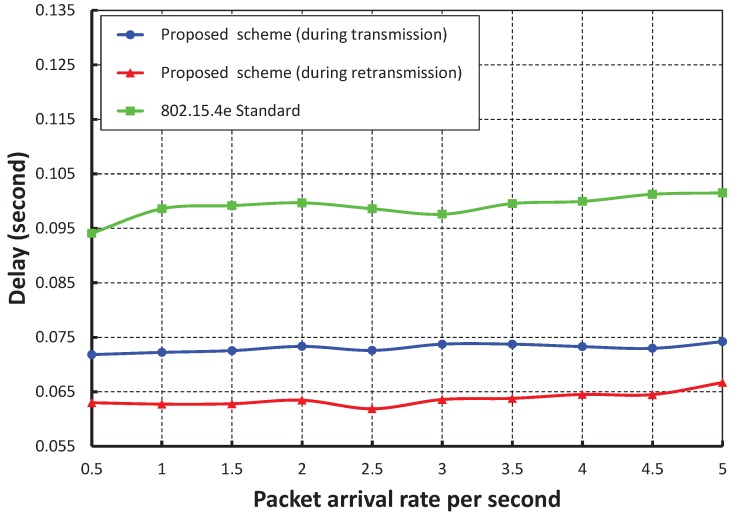
Comparison of the delay of our proposed scheme with the IEEE 802.15.4e standard.

As shown in Figure 15, the number of devices is taken along the *X* axis, and the corresponding delay is obtained as shown along the *Y* axis. It is observed that the delay increases slowly as the number of devices increases when we consider the packet rate as one packet per second. When the data payload is 100 bytes and five devices are considered, the delay is nearly 0.056 s during the transmission and 0.059 s during retransmission, if the packet arrival rate is one packet per second. From Figure 16, it is also observed that the packet transmission delay in our work is significantly less as compared to the IEEE 802.15.4e scheme for different arrival rates, and our simulation result coincides with the analytical one.

**Figure 17:**
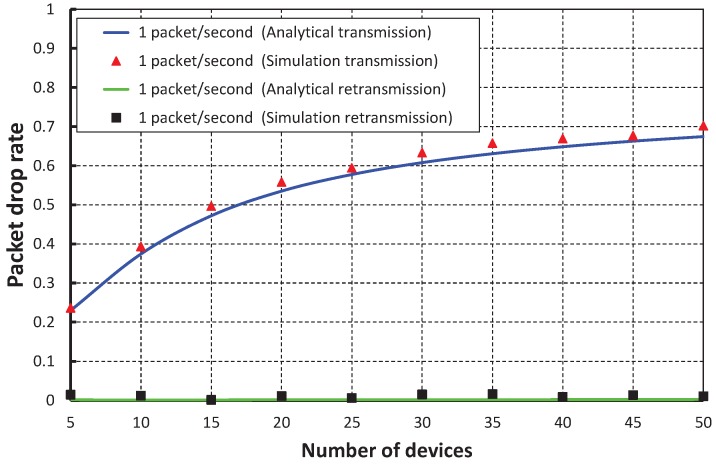
Validation of the packet drop rate of our proposed scheme for different numbers of devices.

**Figure 18:**
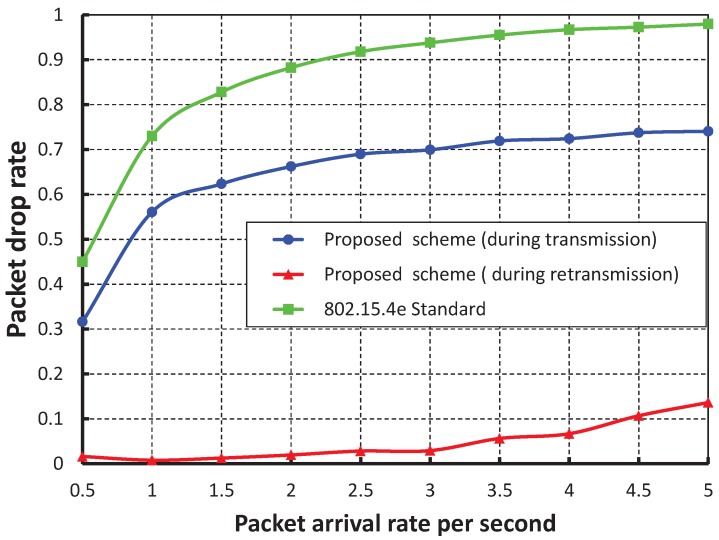
Comparison of the packet drop of our proposed scheme with the IEEE 802.15.4e standard.

Figure 17 shows the packet drop rate with the variable number of devices. It is observed that the packet drop rate increases as the of number of devices increases considering one packet per second. When the data payload is 100 bytes and five devices are considered, the packet drop rate is nearly 0.22 during the transmission. However, the packet drop rate is negligible during the retransmission. As shown in Figure 18, the packet drop rate of our work is significantly less as compared to the IEEE 802.15.4e scheme.

**Figure 19:**
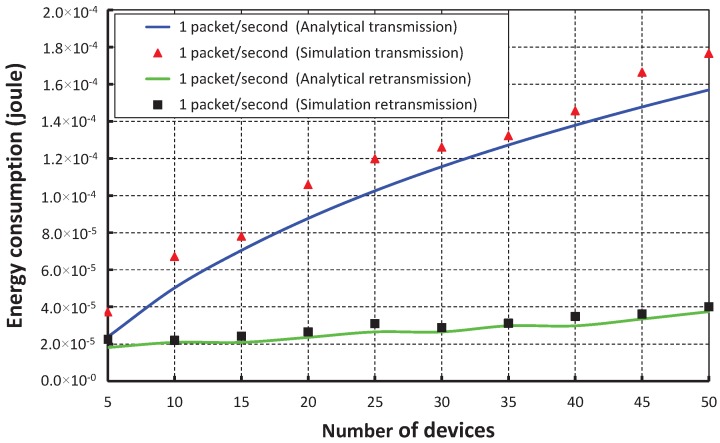
Validation of the energy consumption of our proposed scheme for different numbers of devices.

**Figure 20:**
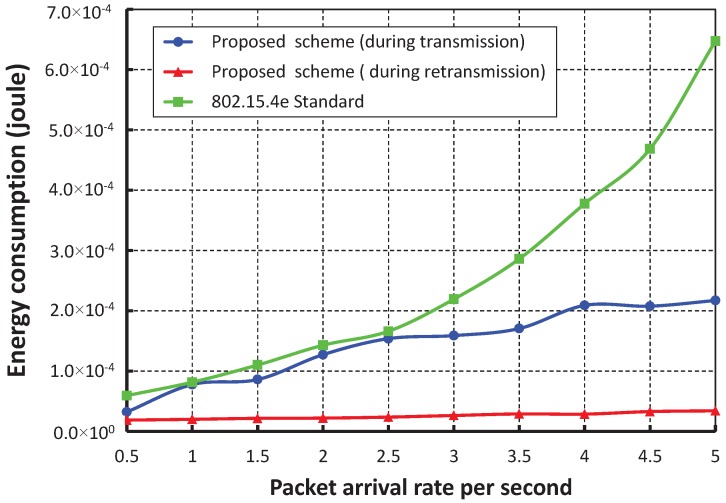
Comparison of the energy consumption of our proposed scheme with the IEEE 802.15.4e standard.

The energy consumption in joules/byte with different numbers of devices when one packet per second arrives at a device is shown in Figure 19. The energy consumption increases as the number device increases. When we consider only five devices and the data payload is 100 bytes, the average energy consumption for successful packet transmission is nearly $2.38466\times 10^{-5}$ Joule during the transmission and $1.81308\times 10^{-5}$ Joule during the retransmission. The energy consumption of our work is significantly less than the IEEE 802.15.4e scheme and is shown in Figure 20. We achieve this improved result through our proposed separate retransmission period and single CCA operation rather than two times consecutive CCA operations as suggested in the IEEE 802.15.4e standard and by allowing only collided devices to contend during the retransmission period.

**Figure 21:**
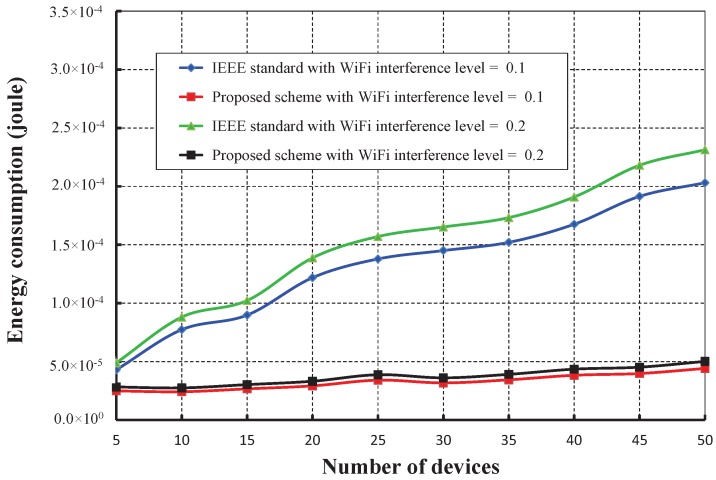
Comparison of the energy consumption of our proposed scheme with the IEEE 802.15.4e standard under different WiFi interference levels.

**Figure 22:**
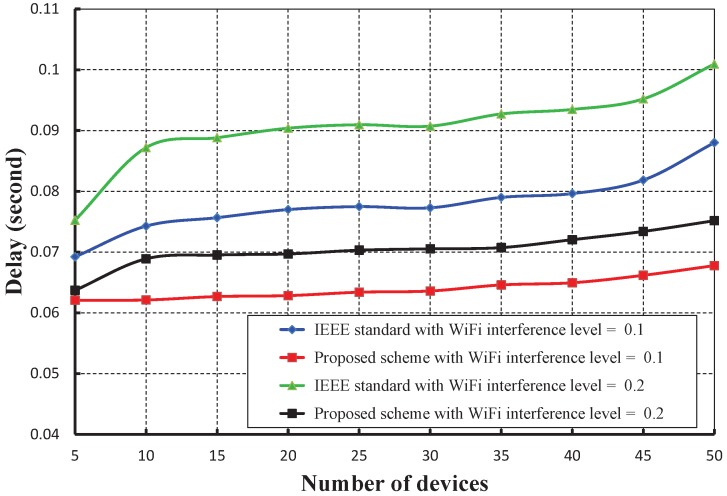
Comparison of the delay of our scheme with the IEEE 802.15.4e standard under different WiFi interference levels.

As shown in Figure 21, the number of devices is taken along the *X* axis, and the corresponding energy consumption in joules is obtained as given along the *Y* axis. It is observed that the energy consumption increases as the numbers of devices attached to a coordinator increase. However, the energy consumption in our schemes is significantly less for different interference levels of WiFi as compared to the IEEE 802.15.4e. Figure 22 shows the delay with a variable number of devices under WiFi interferences. It is observed that the delay increases as the numbers of devices increase considering one packet per second. However, the delay of our mechanism is significantly less as compared to the IEEE 802.15.4e scheme.

**Figure 23:**
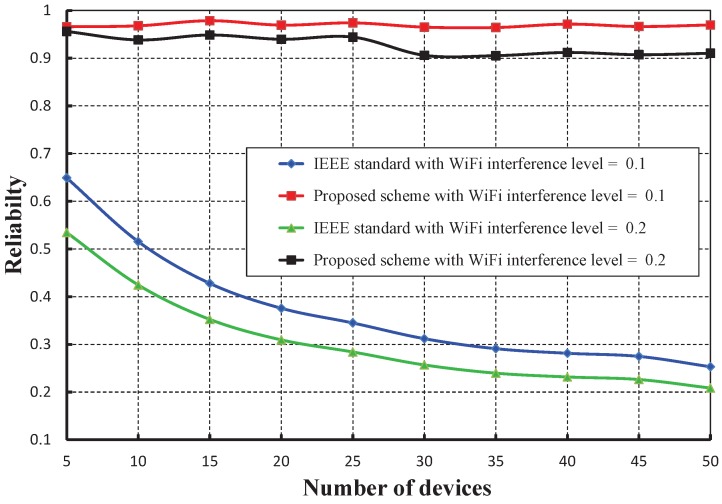
Comparison of the reliability of our proposed scheme with the IEEE 802.15.4e standard under different WiFi interference levels.

**Figure 24:**
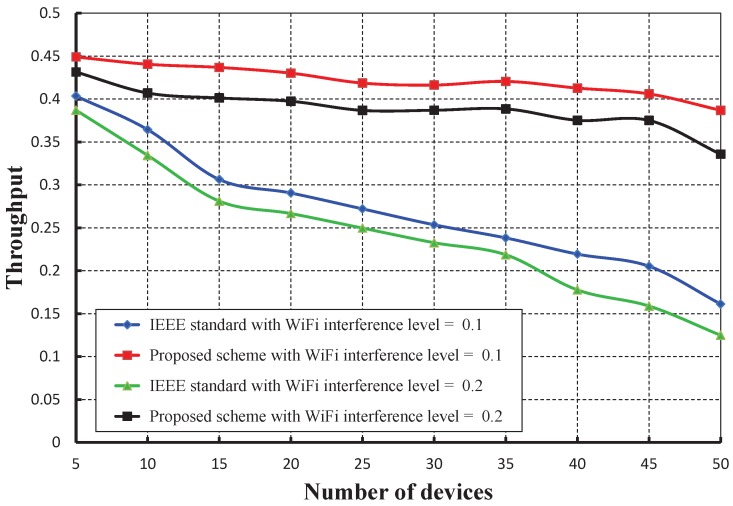
Comparison of the throughput of our proposed scheme with the IEEE 802.15.4e standard under different WiFi interference levels.

Figure 23 compares the reliability of data transmission with respect to different numbers of devices under WiFi interferences. It is observed that the reliability of our work is significantly higher than the IEEE 802.15.4e scheme. The throughput corresponding to different numbers of devices is depicted in Figure 24, and it is observed that the throughput decreases as the number of devices attached to a PAN coordinator increases irrespective of the interference level. It is observed that the throughput of our work is marginally higher than the IEEE 802.15.4e scheme.

## 8. Conclusions

In this paper, a newly-designed superframe structure is proposed for the IEEE 802.15.4e-enabled sensor nodes, where dynamic allocation is made for the transmission failure devices to increase the reliability and reduce the delay. A new data frame structure is proposed to identify the devices due to collision in the hidden terminal problem. A new channel access mechanism is designed based on the current buffer status of the devices, which reduces the packet drop rates and enhances the throughput. Analytical models are developed to study the performance metrics of the network such as reliability, throughput and delay. Our proposed method increases the reliability, throughput and delay significantly of the collided packets. The results obtained from the analytical model and simulation indicate that our mechanisms can improve the performance of the network even if in hidden terminal problems. Hence, our performance analysis models and mechanisms can be implemented in wireless sensor networks for smart home applications, where latency and reliability are the major requirements. 

## Figures and Tables

**Table 1:** Notation table.

Notation	Value
*N*	Number of devices attached to PAN coordinator;
$p_{1}$	Packet generation probability;
σ	Unit backoff slot duration;
$p_{2}$	Probability to defer transmission;
ϕ	Probability of channel access for first time;
α	Probability of channel busy during CCA;
$P_{b}\left(\zeta \right)$	Probability of bit error rate;
$P_{s}$	Probability of transmission attempt successful;
$T_{cca}$	Time duration for CCA;
$T_{pkt}$	Average data packet transmission duration;

**Table 2:** Simulation parameters.

Notation	Value
Radio band	2.4 GHz
Data rate	250 kbps
Carrier sensing sensitivity	−85 dBm
Communication channel number	11
Beacon interval	$15.6\times 2^{5}$ ms
Superframe duration	$15.6\times 2^{3}$ ms
Unit backoff duration	20 symbols
PHY overhead	6 bytes
MAC overhead	3 bytes
Transmission energy consumption	9.1 mA
Receiving energy consumption	5.9 mA
Turnaround energy consumption	7.5 mA
